# Pentacoordinate and Hexacoordinate Mn(III) Complexes of Tetradentate Schiff-Base Ligands Containing Tetracyanidoplatinate(II) Bridges and Revealing Uniaxial Magnetic Anisotropy

**DOI:** 10.3390/molecules21121681

**Published:** 2016-12-08

**Authors:** Ivan Nemec, Radovan Herchel, Zdeněk Trávníček

**Affiliations:** Department of Inorganic Chemistry, Faculty of Science, Palacký University, 17. listopadu 12, CZ-771 46 Olomouc, Czech Republic; ivan.nemec@upol.cz (I.N.); radovan.herchel@upol.cz (R.H.)

**Keywords:** manganese(III), platinum(II), zero-field splitting, magnetic anisotropy, crystal structures

## Abstract

Crystal structures and magnetic properties of polymeric and trinuclear heterobimetallic Mn^III^···Pt^II^···Mn^III^ coordination compounds, prepared from the Ba[Pt(CN)_4_] and [Mn(L4A/B)(Cl)] (**1a**/**b**) precursor complexes, are reported. The polymeric complex [{Mn(L4A)}_2_{μ^4^-Pt(CN)_4_}]_n_ (**2a**), where H_2_L4A = *N*,*N*’-ethylene-bis(salicylideneiminate), comprises the {Mn(L4A)} moieties covalently connected through the [Pt(CN)_4_]^2−^ bridges, thus forming a square-grid polymeric structure with the hexacoordinate Mn^III^ atoms. The trinuclear complex [{Mn(L4B)}_2_{μ-Pt(CN)_4_}] (**2b**), where H_2_L4B = *N*,*N*’-benzene-bis(4-aminodiethylene-salicylideneiminate), consists of two [{Mn(L4B)} moieties, involving pentacoordinate Mn^III^ atoms, bridged through the tetracyanidoplatinate (II) bridges to which they are coordinated in a *trans* fashion. Both complexes possess uniaxial type of magnetic anisotropy, with *D* (the axial parameter of zero-field splitting) = −3.7(1) in **2a** and −2.2(1) cm^−1^ in **2b**. Furthermore, the parameters of magnetic anisotropy **2a** and **2b** were also thoroughly studied by theoretical complete active space self-consistent field (CASSCF) methods, which revealed that the former is much more sensitive to the ligand field strength of the axial ligands.

## 1. Introduction

Single-molecule magnets (SMMs) are compounds composed of the individual molecules capable of preserving their magnetic moment even after removing external magnetic field. This is possible due to an existence of energy barrier ∆ separating the states (formed by the crystal-field splitting of the ground spin state *S*, *S* > 1/2) with minimal and maximal *M*_S_ under the condition of axial magnetic anisotropy. The potential applications of SMMs and their polymeric analogues, so called single-chain magnets (SCMs), include information processing, data storage, quantum computing, spintronics, or biomedical applications [1,2].

**Scheme 1:**
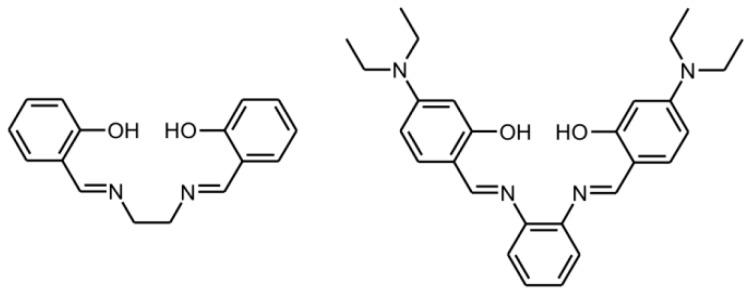
Schematic representations of the tetradentate Schiff base ligands: H_2_L4A (**left**), H_2_L4B (**right**).

Bearing in mind the above mentioned conditions for SMM occurrence it is clear why so much of attention has been devoted to the research of the Mn^III^ complexes involving tetradentate salen-type Schiff base ligands (e.g., L4A^2−^ = salen^2−^ = *N*,*N*’-ethylene-bis(salicylideneiminate) dianion; variously substituted salen ligands will be further abbreviated as H_2_L4). In compounds involving the [Mn(L4)]^+^ moieties the central Mn^III^ atom is in the hexacoordinate environment and, thus, it is the object of the Jahn-Teller effect (*S* = 2, ^5^E crystal field ground term in weak ligand fields). This implies distortion of the coordination polyhedron (its prolongation or compression), which gives rise to significant magnetic anisotropy. These might be of axial (prolongation) or easy-plane (compression) character [3]. Thus, it is clear that compounds of this type are attractive building blocks for synthesis of 0D, 1D, 2D, or 3D coordination compounds exhibiting magnetic bistability [4,5]. A typical approach for synthesis of polymeric or polynuclear coordination compounds involving the [Mn(L4)]^+^ moieties is associated with the reaction of the halide precursor complexes [Mn(L4)X], where X = the halido ligand, with various cyanidometallates. Such reactions lead to preparations of compounds related to Prussian blue [6,7,8,9,10,11]. Previously, we reported on crystal structures and magnetic properties of polymeric and polynuclear compounds involving the [Mn(L4)]^+^ moieties which were bridged by [Fe(CN)_5_(NO)]^2−^, [Pt(SCN)_4_]^2−^ and [Pt(SCN)_6_]^2^ complex anions [12,13,14]. This work represents a continuation of our ongoing study of platinum cyanido/thiocyanido bridged heterometallic compounds and reports on two new Mn^III^···Pt^II^ compounds involving the [Pt(CN)_4_]^2−^ bridges: i.e., [{Mn(L4A)}_2_{*µ*_4_-Pt(CN)_4_}]*_n_* (**2a**) and [{Mn(L4B)}_2_{*µ*-Pt(CN)_4_}] (**2b**), where H_2_L4B = *N*,*N*’-benzene-bis(4-aminodiethylenesalicylideneiminate) (Scheme 1). The prepared complexes were characterized by elemental analysis, molar conductivity, infrared spectroscopy, single crystal X-ray analysis, and magnetic measurements. To the best of our knowledge this is the first report on the crystal structures and magnetic properties of Mn^III^···Pt^II^···Mn^III^ compounds involving [Pt(CN)_4_]^2−^ as bridging units. Furthermore, the CASSCF/NEVPT2 calculations were performed on **2a** and **2b** and also on other model hexa/penta-coordinate compounds with the aim to more deeply understand variations in zero-field splitting (ZFS) parameters in Mn^III^ Schiff-base complexes.

## 2. Results

### 2.1. Crystal Structures

**Figure 1:**
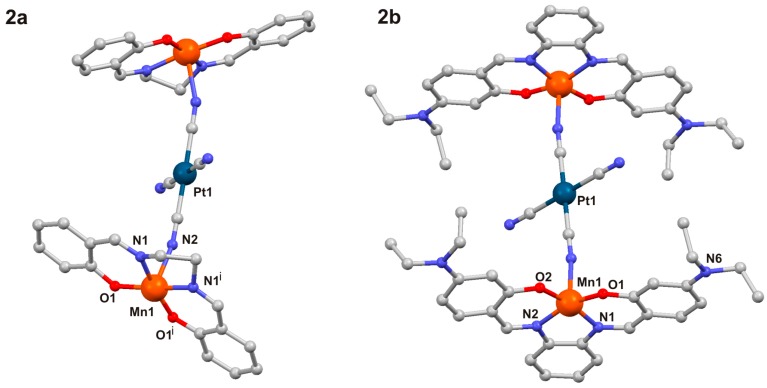
Part of crystal structure of **2a** (**left**) and molecular structure of **2b** (**right**). Hydrogen atoms are omitted due to clarity. Selected bond lengths (in Å) and angles (°): **2a**: Mn1−N1 = 1.963(9), Mn1–N2 = 2.279(8), Mn1−O1 = 1.888(6), O1–Mn1–N1 = 173.1(3), N2–Mn1–N2^i^ = 174.0(4), [Symmetry code: (i) −y, −x, −z + 1/2]; **2b**: Mn1–N1 = 1.974(3), Mn1–N2 = 1.976(3), Mn1–N3 = 2.192(4), Mn1–O1 = 1.879(3), Mn1–O2 = 1.868(3), O1–Mn1–N2 = 168.97(13), O2–Mn1–N1 = 161.89(14).

The crystal structures of **2a** and **2b** were determined by single-crystal X-ray analysis (Figure 1, Table 1). Both compounds comprise Pt^II^ atoms coordinated by four carbon atoms from the cyanide groups, thus forming a {PtC_4_} chromophore (Figure 1). The Pt−C bond lengths are from the narrow range of 1.99–2.00 Å (Figure 1), and with the C−Pt−C angles close to the ideal straight angle as expected for the square planar chromophore geometry (179.6(5)° in **2a**, and 180° in **2b**). In both compounds, the Schiff base ligands (Scheme 1) coordinate Mn^III^ atoms by two imino nitrogen (N_im_) and two phenolate oxygen atoms with the metal−donor atom distances: Mn–N_im_ = 1.96–1.98 Å and Mn–O = 1.86–1.89 Å.

**Figure 2:**
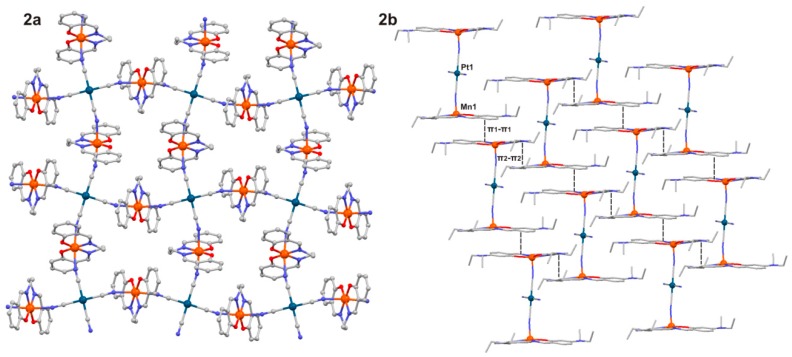
Fragments of the crystal structures of the complexes **2a** and **2b**. The hydrogen atoms are omitted for clarity. Several *π*–*π* stacking are displayed in the complex **2b** (black dashed lines).

Crystal structure of **2a** is isostructural to the previously reported compound [Mn(L4A)}{*µ*^4^-Ni(CN)_4_}]_n_ [15]. The N_im_ and O donor atoms form the equatorial plane of the coordination polyhedron of the Mn^III^ center, while the axial coordination sites are occupied by two symmetrically equivalent nitrogen atoms (N_CN_) from bridging [Pt(CN)_4_]^2−^ anions. Thus, the Mn^III^ atom is hexacoordinate with the {MnN_2_N’_2_O_2_} chromophore. The Mn–N_CN_ bond lengths are significantly longer than the Mn–N_im_ bonds due to a Jahn-Teller distortion: Mn–N_CN_ = 2.279(8) Å. Each [Pt(CN)_4_]^2−^ bridging anion bonds to four [Mn(L4A)]^+^ moieties forming thus a square grid architecture and therefore, the overall crystal structure of **2a** can be classified as a two-dimensional polymer (Figure 2). The neighboring [Mn(L4A){*µ*^4^-Pt(CN)_4_}]_n_ layers are weakly interconnected by the offset *π*−*π* stacking [16,17,18] of aromatic rings (the shortest C···C distance is 3.319(7) Å).

In **2b**, the *trans*-cyanido ligands of the [Pt(CN)_4_]^2−^ bridges bond to the Mn^III^ atoms, thus forming trinuclear [{Mn(L4B)}{*µ*-Pt(CN)_4_}] complex molecules (Figure 1). One axial position on each Mn^III^ center is unoccupied and, thus, the chromophore is pentacoordinate {MnN_2_N’O_2_} with the coordination geometry close to ideal square pyramidal (*τ* = 0.12) [19]. The Mn−N_CN_ distance in **2b** (2.193(4) Å) is significantly shorter than in **2a** (2.279(3) Å).

The reason for such difference is that the pentacoordinate Mn^III^ center in **2b** is not affected by Jahn-Teller effect. Furthermore, it must be stressed that the Mn^III^ atom is involved into metal···*π* non-covalent interactions with carbon atoms from aromatic part of the L4B^2−^ ligands from the adjacent trinuclear complex molecules (Figure 2). The Mn···C distances in such non-covalent contact are shorter (3.423(4) and 3.449(4) Å) than the sum of their van der Waals radii (3.7 Å) [20]. Other non-covalent interactions presented in **2b** are of *π*–*π* stacking nature. They do extend the crystal structure of **2b** to 2D supramolecular layers (Figure 2). Two different stacking interactions can be distinguished: (a) intrachain, *π*_1_−*π*_1_, with the shortest C···C distance at 3.338(5) Å, and (b) interchain, *π*_2_*−π*_2_, with the shortest C···C distance of 3.403(5) Å (Figure 2).

### 2.2. Magnetic Properties

**Figure 3:**
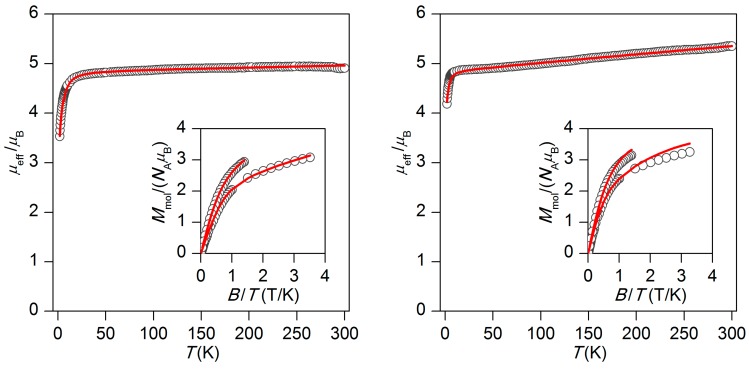
The magnetic data for **2a** (**left**) and **2b** (**right**) showing the temperature dependence of the effective magnetic moment calculated from the molar magnetization measured at *B* = 0.1 T, and the reduced isothermal magnetizations measured at *T* = 2 and 5 K. Empty circles—experimental data, full lines—calculated data, using Equation (1), and *D* = −3.7(1) cm^−1^, *g* = 1.981(6), *zj* = −0.244(9) cm^−1^, *χ*_TIM_ = 6(1)·10^−9^ m^3^·mol^−1^ for **2a** and *D* = −2.2(1) cm^−1^, *g* = 1.975(8), *zj* = −0.02(1) cm^−1^, *χ*_TIM_ = 28(2) 10^−9^ m^3^·mol^−1^ for **2b**.

The magnetic data of complexes **2a** and **2b** are depicted in Figure 3 and conform with practically isolated paramagnetic Mn^III^ cations. The effective magnetic moment is linearly decreasing from the room temperature value of 4.91 *μ*_B_ to the value of 4.78 *μ*_B_ at 30 K for **2a**, and from 5.35 *μ*_B_ to the value of 4.89 *μ*_B_ at 30 K for **2b**. The decrease of the effective magnetic moment in this temperature region is much more apparent for **2b** and this can be ascribed to a presence of small amount of unknown magnetic impurity undetectable by other physical methods. Then, the effective magnetic moment drops to the value of 3.53 *μ*_B_ for **2a** and to the value of 4.19 *μ*_B_ for **2b** at the lowest available temperature (*T* = 1.9 K). This is mainly due to the zero-field splitting (ZFS) of Mn^III^ atoms and partly also due to very weak intra/inter-molecular non-covalent interactions. Therefore, we postulated the following spin Hamiltonian:

(1) $$\hat{H}=D\left({\hat{S}}_{z}^{2}-{\hat{S}}^{2}/3\right)+\mu_{B}Bg{\hat{S}}_{a}-zj\langle {\hat{S}}_{a}\rangle {\hat{S}}_{a}$$

where the first term describes the magnetic anisotropy with single-ion axial ZFS parameter *D*, the second term is the Zeeman term, and the last term, represented with the *zj* variable, is the common molecular-field correction parameter, which is due to intermolecular interactions. The <*S_a_*> is a thermal average of the molecular spin projection in the *a* direction of magnetic field defined as *B_a_* = *B*∙(sin*θ*cos*ϕ*, sin*θ*sin*ϕ*, cos*θ*) with the help of polar coordinates. Then, the molar magnetization in the *a*-direction of magnetic field can be numerically calculated as:

(2) $$M_{a}=-N_{A}\frac{\sum_{i}\left(\sum_{k}\sum_{l}C_{ik}^{+}\left(Z_{a}\right)C_{li}\right)\exp\left(-\varepsilon_{a,i}/kT\right)}{\sum_{i}\exp\left(-\varepsilon_{a,i}/kT\right)}$$

where *Z_a_* is the matrix element of the Zeeman term for the *a-*direction of the magnetic field and *C* are the eigenvectors resulting from the diagonalization of the complete spin Hamiltonian matrix. The presence of *zj* means that iterative procedure must be used [21]. Then, the averaged molar magnetization of the powder sample was calculated as integral (orientational) average:

(3) $$M_{\mathrm{mol}}=1/4\pi \int_{0}^{2\pi }\int_{0}^{\pi }M_{a}\sin\theta d\theta d\varphi$$

In order to obtain reliable parameters, both temperature and field dependent magnetic data were fitted simultaneously, which resulted in these parameters: *D* = −3.7(1) cm^−1^, *g* = 1.981(6), *zj* = −0.244(9) cm^−1^, *χ*_TIM_ = 6(1)·10^−9^ m^3^·mol^−1^ for **2a**, and *D* = −2.2(1) cm^−1^, *g* = 1.975(8), *zj* = −0.02(1) cm^−1^, *χ*_TIM_ = 28(2)·10^−9^m^3^·mol^−1^ for **2b**, where the temperature-independent magnetism correction *χ*_TIM_ was applied to describe the contribution of traces of magnetic impurities [22]. The sign of *D*-parameters suggests that in **2a** and **2b** there is axial magnetic anisotropy and that more negative *D* value found for **2a** revealed larger anisotropy in the hexacoordinate Mn^III^ compound as compared to that of pentacoordinate one.

### 2.3. Theorethical Calculations

Furthermore, we supported our experimental magnetic study also by ab initio CASSCF/NEVPT2 calculations using ORCA computational package where also the relativistic effects were included as described in the Section 4.2.3. The resulting values of ZFS parameters were as follows: *D* = −3.57 cm^−1^ and *E*/*D* = 0.030 for **2a**, and *D* = −2.75 cm^−1^ and *E*/*D* = 0.021 for **2b**, while the components of *g*-tensors for both compounds were in narrow range from 1.977 to 1.994. Thus, the calculations supported our conclusions following from the experimental results showing that there is lower magnetic anisotropy in the pentacoordinate complex **2b** than in the hexacoordinate one (**2a**).

## 3. Discussion

**Figure 4:**
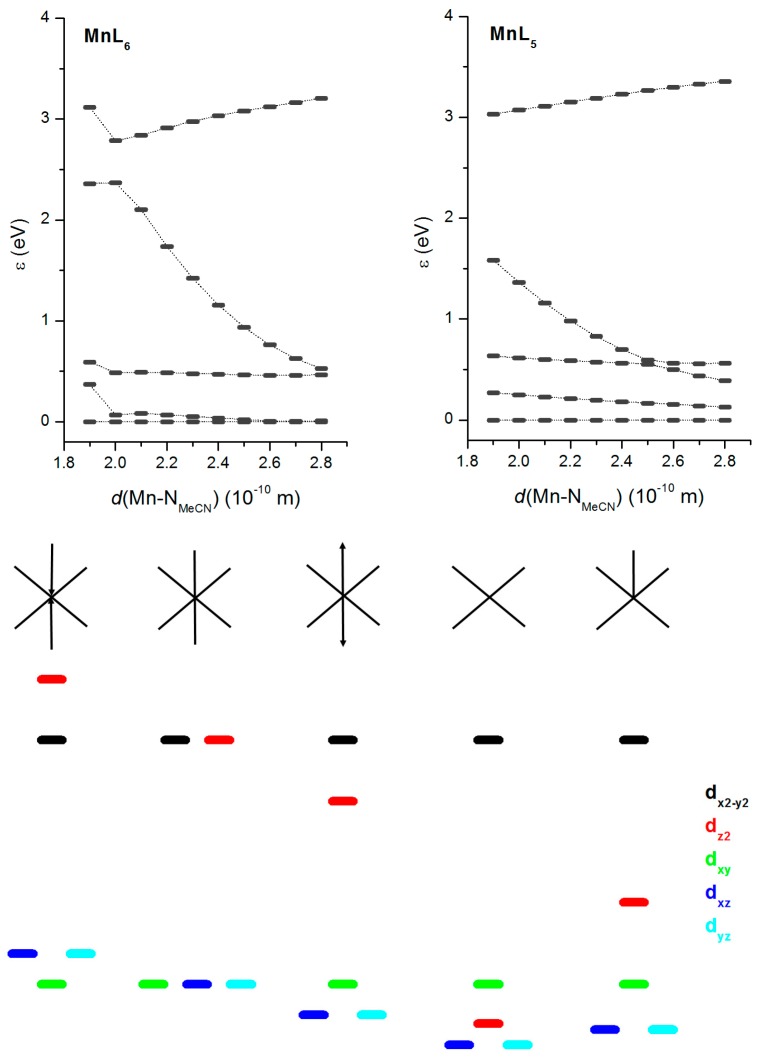
**Top**: the calculated *d*-orbitals energy splitting for the model compounds [Mn(L4A)(MeCN)]^+^ (MnL_6_) and [Mn(L4A)(MeCN)_2_]^+^ (MnL_5_) for varying Mn–N_MeCN_ distance(s) from 1.9–2.8 Å using B3LYP/ZORA/def2-TZVP(-f); **Bottom**: variation of *d*-orbitals energy according to the crystal-field theory for idealized geometries (compressed square-bipyramid, octahedral, elongated square-bipyramid, square-planar, and square-pyramidal).

**Figure 5:**
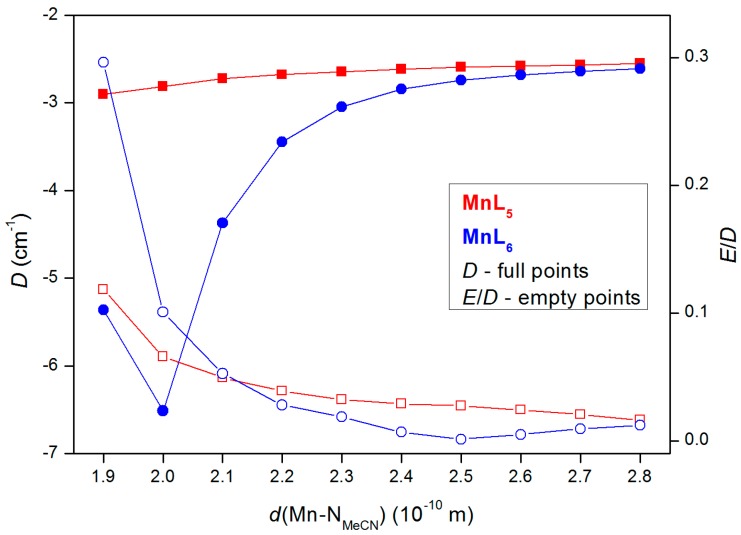
The CASSCF/NEVPT2 calculated magneto-structural correlation of ZFS parameters *D* and *E* for pentacoordinate [Mn(L4A)(MeCN)]^+^ (MnL_5_) and hexacoordinate [Mn(L4A)(MeCN)_2_]^+^ (MnL_6_) model compounds for varying Mn–N_MeCN_ distance(s) from 1.9–2.8 Å.

In order to improve our understanding of magnetic anisotropy in these complexes, we performed magneto-structural correlations for pentacoordinate [Mn(L4A)(MeCN)]^+^ (MnL_5_) and hexacoordinate [Mn(L4A)(MeCN)_2_]^+^ (MnL_6_) model compounds, where we studied the impact of axial ligand(s) field strength on ZFS parameters by varying Mn−N_MeCN_ distance(s) from 1.9 to 2.8 Å (MeCN = acetonitrile). The geometries of the complexes were optimized with the PBE functional also incorporating the COSMO model (COSMO = COnductor-like Screening MOdel). The only geometrical constrain applied was Mn–N_MeCN_ distance and in case of hexacoordinate MnL6 model compound, the Mn–N_MeCN_ distances were both equal. Subsequently, the single-point energy calculations were done for each optimized geometry using B3LYP/ZORA/def2-TZVP(-f) followed by extracting the information about the splitting of *d*-orbitals, which is visualized in Figure 4 (top). This figure resembles the well-known crystal-field theory schemes outlined also in Figure 4 (bottom). In the case of hexacoordinate MnL_6_ model compound with *d*(Mn–N_MeCN_) = 1.9 Å, the d-orbitals are evidently split according to scheme outlined for a compressed square-bipyramidal coordination geometry, whereas for increasing Mn–N_MeCN_ distance, the pattern for elongated square-bipyramidal arrangement is observed. Upon further increase in the Mn–N_MeCN_ distance, the d-orbitals are split similarly to square-planar geometry. An analogous situation can be found for the pentacoordinate MnL_5_ model compound, where an increase in the Mn–N_MeCN_ distance follows d-orbitals splitting from square-pyramidal to square-planar geometry. Of course, DFT-calculated (DFT = Density Functional Theory) energies of *d*-orbitals are not exactly following simplified schemes derived from the crystal-field theory due to non-equivalent ligand field strengths of Schiff base nitrogen and oxygen donor atoms. Then, ZFS parameters for each geometry were calculated by the same procedure with the CASSCF/NEVPT2 method. The results are depicted in Figure 5.

**Figure 6:**
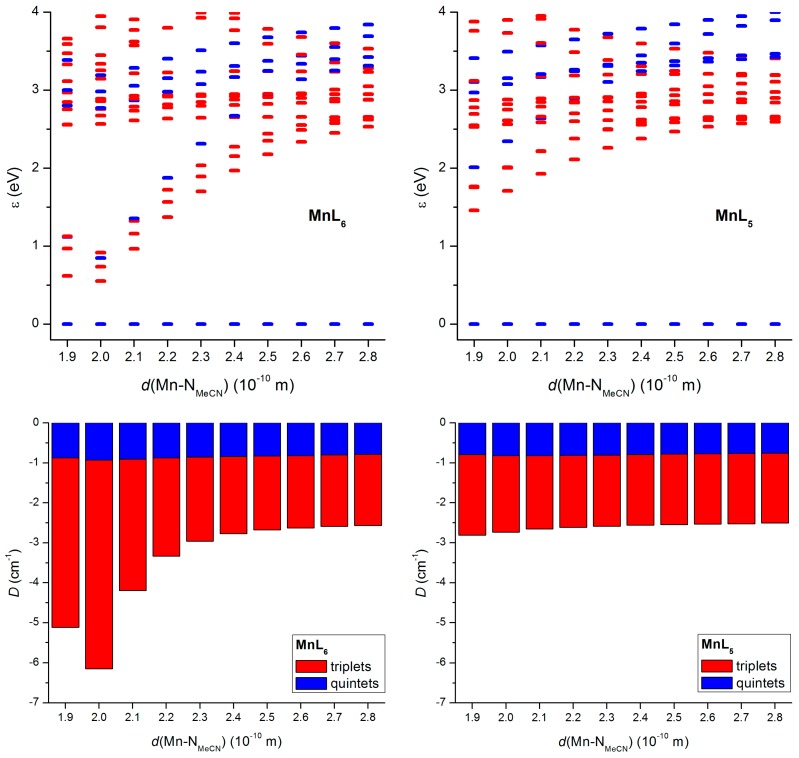
**Top**: ligand field terms arising from CASSCF/NEVPT2 calculations for the model compounds [Mn(L4A)(MeCN)]+ (MnL_5_) and [Mn(L4A)(MeCN)_2_]^+^ (MnL_6_) done for varying Mn–N_MeCN_ distance(s) from 1.9–2.8 Å. Quintet states are blue and triplet states are red; **Bottom**: the contribution of quintet (**blue**) and triplet (**red**) states to the calculated *D*-parameter.

It is evident that there is a negligible effect of the axial ligand strength on magnetic anisotropy in the case of the pentacoordinate MnL_5_ complex, the *D* varied only between −2.9 and −2.5 cm^−1^. This is in the stark contrast with the hexacoordinate MnL_6_ complex, where shortening of the Mn−N distances (axial elongation → nearly ideal octahedron), in other words, increasing the ligand field strength of the axial ligands, led to immense increase of the negative value of the *D*-parameter (*D* = −2.6 → −6.5 cm^−1^) with the maximal absolute value at the chromophore geometry close to the ideal octahedral arrangement (Figure 5). Further increase in the axial ligand field strength (i.e., the axial compression) would lead to crossover to compressed square-bipyramid and consequently to a positive value of the *D*-parameter, which is evidenced here by increase of *E*/*D* ratio approaching the value of 1/3 (Figure 5). Such a relationship between the sign of *D* and the axial elongation/compression was already proposed by Maurice et al. for a simple [Mn(NCH)_6_]^3+^ model complex [23]. Furthermore, we plotted the ligand field terms, quintets and triplets, arising from CASSCF/NEVPT2 calculations for both MnL_5_ and MnL_6_ model compounds (Figure 6). It is evident that the increase in the axial ligand field strength induced by decreasing of Mn–N_MeCN_ distances led to significant lowering of the excited quintets and especially triplet states for MnL_6_, whereas energies of excited triplets and quintets states are not varied to such extent for MnL_5_. This different behaviour had dominant impact on the value of the *D*-parameter, because the contribution of quintet states to *D* is more or less the same for all Mn–N_MeCN_ distances and moreover for both hexa- and pentacoordinate model complexes (Figure 6). Furthermore, the comparison of *D*-values for MnL_6_ and MnL_5_ model compounds at *d*(Mn–N_MeCN_) = 2.2–2.3 Å (Figure 5), that means for axial Mn–N distances found in **2a** and **2b**, clearly showed that more negative *D* is expected for hexacoordinate compound as also confirmed from the analysis of the experimental magnetic data of the reported compounds.

## 4. Materials and Methods

### 4.1. Materials

All of the starting chemicals were of analytical reagent grade and were used as received. All of the chemicals were purchased from commercial sources (Sigma Aldrich, St. Louis, MO, USA).

#### 4.1.1. Preparation of Ba[Pt(CN)_4_]

A solution of PtCl_2_ (1 mmol, in 10 mL of water) was added to a cold, saturated solution of KCN (4 mmol, in 2 mL of water). The precipitated K_2_[Pt(CN)_4_]·3H_2_O was filtered off. To the solution of K_2_[Pt(CN)_4_]·3H_2_O (1 mmol, in 5 mL of water) the solution of BaCl_2_ (1 mmol, in 3 mL of water) was added. The resulting green precipitate was filtered off, washed with diethyl ether and stored in a desiccator. Yield: 89%. Elem. anal. Calcd (%) for C_4_N_4_Ba_1_Pt_1_: C, 11.00; N, 12.83. Found: C, 11.33; N, 12.65. FT-IR data (ν_max_ (ATRd)/cm^−1^): 3512 w; 3270 w; 2653 w; 2596 w; 2286 w; 2120 vs ν(C≡N); 1982 w; 1653 w; 1618 m; 733; 561 w.

#### 4.1.2. Preparation of [{Mn(L4A)}_2_{μ^4^-Pt(CN)_4_}]_n_ (**2a**), and [{Mn(L4B)}_2_{μ-Pt(CN)_4_}] (**2b**)

The precursor complexes [Mn(L4A)Cl], **1a**, and [Mn(L4B)Cl], **1b**, were prepared according to the procedures in the literature [24].

A water solution (10 mL) of Ba[Pt(CN)_4_] (0.1 mmol) was added under continuous stirring to a methanol solution (10 mL) of [Mn(L4A/B)Cl] **1a/b** (0.2 mmol). The reaction mixture was stirred for 60 min at room temperature, and then was kept undisturbed in the dark. After twelve days, single-crystals suitable for X-ray analysis were formed. The resulting crystals were filtered off from the mother liquor, washed with water, diethyl ether, dried in a drying kiln (at 50 °C), and stored in a desiccator.

**2a**: Yield: 85%. Elem. anal. Calcd (%) for C_36_H_28_N_8_O_4_Mn_2_Pt_1_: C, 45.91; H, 2.99; N, 11.90. Found: C, 45.86; H, 2.74; N, 12.26. FT-IR (ATR/cm^−1^): 594 m; 588 m; 550 w; 502 w; 456 s; 433 m; 379 s; 353 m; 341 m; 318 m; 296 m ν(Mn−N); 247 w ν(Mn−O); 210 w. FT-IR (KBr/cm^−1^): 3103 w; 3044 w ν(C−H)_ar_; 2958 w ν(C−H)_alip_; 2952 w ν(C−H)_alip_; 2626 w; 2142s ν(C≡N); 1982 w; 1625 vs ν(C=N)_ar_; 1596 m, 1538 m, 1465 m ν(C=C)_ar_; 1441 m; 1389 w; 1328 w; 1287 m; 1238 w; 1199 w; 1146 w; 1127 w; 1084 w; 1049 w; 1029 w; 901 w; 862 w; 795 w; 764 w; 647 w.

**2b**: Yield: 81%. Elem. anal. Calcd (%) for C_60_H_64_N_12_O_4_Mn_2_Pt_1_: C, 54.50; H, 4.87; N, 12.71. Found: C, 54.71; H, 4.68; N, 12.89. FT-IR (ATR/cm^−1^): 577 w; 538 w; 529 w; 520 w; 499 w; 482 w; 462 w; 438 w; 421 w; 400 w; 355 w; 345 w; 316 w; 288 w, 258 w. FT-IR data (KBr/cm^−1^): 3288 w; 3078 w ν(C−H)_ar_; 2972 w ν(C−H)_alip_; 2229 w ν(C−H)_alip_; 2757 w; 2142 s ν(C≡N); 1609s ν(C=N)_ar_; 1559 m, 1516 m, 1486 m ν(C=C)_ar_; 1431 w; 1412 w; 1374 w; 1334 w; 1310 w; 1276 w; 1244 m; 1208 w; 1189 w; 1170 w; 1139 w; 1076 w; 1039 w; 1019 w; 965 w; 825 w; 787 m; 741 m; 701 w; 650 w.

### 4.2. Methods

#### 4.2.1. General Methods

Elemental analysis (CHN) was performed on a FLASH 2000 CHN Analyser (ThermoFisher Scientific, Waltham, MA, USA). Infrared spectra of the complexes were recorded on a NEXUS 670 FT-IR spectrometer (ThermoNicolet, Waltham, MA, USA) using the ATR technique on a diamond plate in the range 600–4000 cm^−1^. The reported FT-IR intensities were defined as w = weak, m = medium, s = strong, and vs = very strong. The magnetic data were measured on powdered samples pressed into pellets using a MPMS XL-7 Quantum Design SQUID magnetometer (Quantum Design Inc., San Diego, CA, USA). The experimental data were corrected for the diamagnetism of the constituent atoms by using Pascal’s constants.

#### 4.2.2. Single Crystal X-ray Analysis Details

Single crystal X-ray diffraction data of **2a** and **2b** were collected on an Oxford diffractometer Xcalibur2 (Oxford Diffraction Ltd., Oxford, UK) with the Sapphire CCD detector and fine-focused sealed tube (Mo Kα radiation, *λ* = 0.71073 Å) source and equipped with an Oxford Cryosystem nitrogen gas flow apparatus. All structures were solved by direct methods using SHELXS-2014 [25] incorporated into the WinGX program package [26]. For each structure, its space group was checked by the ADSYMM procedure with PLATON software [27,28]. All structures were refined using full-matrix least-square procedures on *F*^2^ with SHELXL-2014 [25] with anisotropic displacement parameters for all non-hydrogen atoms. The hydrogen atoms were placed into the calculated positions and they were included into the riding model approximation, with *U*_iso_ = 1.2 or 1.5 *U*_eq_. All of the crystal structures were visualized using Mercury software [16].

#### 4.2.3. Theoretical Methods

Ab initio CASSCF/NEVPT2 calculations [29,30] using ORCA 3.0 [31] with active space defined by four electrons in five *d*-atomic orbitals, CAS(4,5) and taking into account five quintets and 45 triplets. The relativistic effects were also included in the calculations with zero order regular approximation (ZORA) [32,33] together with the scalar relativistic contracted version of def2-TZVP(-f) basis functions [34]. The calculations utilized the RI approximation with the decontracted auxiliary def2-TZV/C Coulomb fitting basis sets and the chain-of-spheres (RIJCOSX) approximation to exact exchange [35]. Increased integration grids (Grid5 in ORCA convention) and tight SCF convergence criteria were used. The ZFS parameters, based on dominant spin-orbit coupling contributions from excited states, were calculated through quasi-degenerate perturbation theory (QDPT) [36], in which approximations to the Breit-Pauli form of the spin-orbit coupling operator (Spin-Orbit Mean-Field (SOMF) approximation) [37] and the effective Hamiltonian theory [38] were utilized.

The geometries of pentacoordinate [Mn(L4A)(MeCN)]^+^ and hexacoordinate [Mn(L4A)(MeCN)_2_]^+^ model compounds were optimized with the PBE functional [39] again using the scalar relativistic contracted version of def2-TZVP(-f) basis functions incorporating the COSMO model [40]. The single-point energy calculations were done for each optimized geometry using B3LYP functional [41,42,43]. ZFS parameters were calculated by the same procedure with the CASSCF/NEVPT2 method described above.

## 5. Conclusions

In conclusion, this work presents synthesis and characterization of two new Mn^III^···Pt^II^···Mn^III^ complexes with the general formula [{Mn(L4A)}_2_{*μ*_4_-Pt(CN)_4_}]*_n_* (**2a**) and [{Mn(L4B)}_2_{*μ*-Pt(CN)_4_}] (**2b**). Both compounds were investigated by single crystal X-ray diffraction and their magnetic properties were studied by temperature and field dependent measurements of magnetic moment. These studies revealed that compound **2a** has a two-dimensional polymeric structure with the hexacoordinate Mn^III^ atoms, while **2b** possesses a trimeric structure with both Mn^III^ atoms being pentacoordinate. The analysis of magnetic data showed that the magnetic anisotropy is uniaxial in both compounds, with *D* = −3.7(1) for **2a** and −2.2(1) cm^−1^ for **2b**. These findings were also supported by CASSCF/NEVPT2 calculations (*D* = −3.57 cm^−1^ and *E*/*D* = 0.030 for **2a**, and *D* = −2.75 cm^−1^ and *E*/*D* = 0.021 for **2b**). Further theoretical modelling of other pentacoordinate [Mn(L4A)(MeCN)]^+^ and hexacoordinate [Mn(L4A)(MeCN)_2_]^+^ model compounds elucidated why the axial ZFS parameter *D* is larger in the case of the latter. The next important consequence is that magnetic anisotropy cannot be practically tuned in pentacoordinate square-pyramidal complexes by modifying ligands in axial position. On the contrary, hexacoordinate square-bipyramidal complexes are sensitive to the axial ligand field, thus prone to tuning of magnetic anisotropy, and for that reason they indicate themselves as promising for the preparation of the single-molecule magnets (SMMs).

## Figures and Tables

**Table 1:** Crystal data and structure refinements for **2a** and **2b**.

	2a	2b
Formula	C_36_H_28_Mn_2_N_8_O_4_Pt	C_30_H_32_MnN_6_O_2_Pt_0.50_
Mr	941.62	661.10
Space group	*P*4/*ncc*	*P*-1
Crystal system	tetragonal	triclinic
*a*/Å	14.7755(3)	10.6904(4)
*b*/Å	14.7755(3)	12.6273(3)
*c*/Å	16.7105(5)	13.2804(3)
*α*/°	90	70.773(2)
*β*/°	90	79.588(2)
*γ*/°	90	67.676(3)
*V*/Å^3^	3648.2(2)	1562.69(9)
*T/K*	150(2)	150(2)
*D*_C_/g cm^−3^	1.717	1.405
*μ*/mm^−1^	4.568	2.684
F(000)	1846.0	668.0
Reflections collected/unique	1612/1435	5396/4748
Data/restraints/parameters	1612/1/115	5396/0/361
Goodness of fit (GOF) on F^2^	1.307	1.004
*R*_1_, w*R*_2_ (I > 2*δ*(I)) ^a,b^	0.0500/0.1166	0.0368/0.0817
*R*_1_, w*R*_2_ (all data) ^a,b^	0.0544/0.1166	0.0425/0.0829
CCDC number	1510379	1510378

^a^ *R*_1_ = ∑(|*F*_o_|–|*F*_c_|)/∑|*F*_o_|; ^b^ w*R*_2_ = {∑[w(*F*_o_^2^ – *F*_c_^2^)^2^]/∑[w(*F*_o_^2^)^2^]}^1/2^.
